# Do rocker-sole shoes influence postural stability in chronic low back pain? A randomised trial

**DOI:** 10.1136/bmjsem-2016-000170

**Published:** 2016-10-19

**Authors:** C Sian MacRae, Duncan Critchley, Matthew Morrissey, Adam Shortland, Jeremy S Lewis

**Affiliations:** 1 College of Health and Life Sciences, Brunel University, Uxbridge, UK; 2 Therapy Services, Chelsea and Westminster Hospital NHS Foundation Trust, London, UK; 3 Academic Department of Physiotherapy and Division of Health and Social Care Research, King's College London, London, UK; 4 Faculty of Health Sciences, University of Ljubljana, Ljubljana, Slovenia; 5 Guy's and St Thomas’ NHS Foundation Trust, One Small Step Gait Laboratory, London, UK; 6 Biomedical Engineering, King's College London, London, UK; 7 Department of Allied Health Professions, University of Hertfordshire, Hatfield, UK; 8 Musculoskeletal Services, Central London Community Healthcare NHS Foundation Trust, London, UK

**Keywords:** Biomechanics, Exercise rehabilitation, Lumbar spine, Randomised controlled trial

## Abstract

**Background:**

People with chronic low back pain (CLBP) demonstrate greater postural instability compared with asymptomatic individuals. Rocker-sole shoes are inherently unstable and may serve as an effective balance training device. This study hypothesised that wearing rocker-sole shoes would result in long-term improvement in barefoot postural stability in people with CLBP.

**Methods:**

20 participants with CLBP were randomised to wear rocker-sole or flat-sole shoes for a minimum of 2 hours each day. Participants were assessed barefoot and shod, over three 40 s trials, under 4 posture challenging standing conditions. The primary outcome was postural stability assessed by root mean squared error of centre of pressure (CoP) displacement (CoP_RMSE AP_) and mean CoP velocity (CoP_VELAP_), both in the anteroposterior direction, using force plates. Participants' were assessed without knowledge of group allocation at baseline, 6 weeks and 6 months (main outcome point). Analyses were by intention-to-treat.

**Results:**

At 6 months, data from 11 of 13 (84.6%) of the rocker-sole and 5 of 7 (71.4%) of the flat-sole group were available for analysis. At baseline, there was a mean increase in CoP_RMSE AP_ (6.41 (2.97) mm, p<0.01) and CoP_VELAP_ (4.10 (2.97) mm, p<0.01) in the rocker-sole group when shod compared with barefoot; there was no difference in the flat-sole group. There were no within-group or between-group differences in change in CoP parameters at any time point compared with baseline (1) for any barefoot standing condition (2) when assessed shod eyes-open on firm ground.

**Conclusions:**

Although wearing rocker-sole shoes results in greater postural instability than flat-sole shoes, long-term use of rocker-sole shoes did not appear to influence postural stability in people with CLBP.

**What are the new findings?**

- Standing in a rocker-sole shoe reduced postural stability compared with standing barefoot, whereas standing in a flat-sole shoe did not influence postural stability.
- Long-term use of rocker-sole or flat-sole shoes do not influence postural stability in barefoot standing.

**How might it impact on clinical practice in the near future?**

- This study questions the belief that balance rehabilitation, especially when delivered in standing using rocker-sole shoes, will result in a long-term influence on postural control in people with chronic low back pain (CLBP). Treatment approaches directed towards influencing or ‘normalising’ altered CoP parameters may not be appropriate for people with CLBP.

## Introduction

Differences in postural control during standing have been reported in people with chronic low back pain (CLBP).^1–9^ During more challenging standing conditions, defined as standing on compliant ground with visual occlusion, people with CLBP demonstrate increased centre of pressure (CoP) displacements and velocities, thought to indicate a reduced ability to maintain postural stability.^10^ These differences in postural control have been proposed as underpinning mechanisms in the presence and recurrent nature of CLBP.^7^ ^11^

Greater CoP displacements, interpreted as increased postural instability, are reported during standing wearing rocker-sole compared with traditional flat-sole shoes,^12–14^ suggesting rocker-sole shoes may act as a balance training device. Rehabilitation with proprioceptive or balance training has demonstrated clinical benefits in people with functional ankle instability and anterior cruciate ligament-deficient knees^15^ ^16^ and is recommended as a CLBP treatment.^17^ To the authors' knowledge, no published study has investigated the short-term and long-term influence of rocker-sole shoes on postural stability in people with CLBP. Hence, the following hypotheses were investigated:

- H1:Standing in rocker-sole shoes will promote a greater postural instability than standing in flat-sole shoes in the anteroposterior direction compared with barefoot standing.
- H2:Individuals presenting with CLBP who wear rocker-sole shoes as part of their rehabilitation programme will improve their barefoot standing stability in the anteroposterior direction in the shorter (6 weeks) and longer term (6 months) against those who wear standard flat-sole trainers.

## Methods

This randomised trial with repeated measures recruited participants from a study investigating the influence of footwear on CLBP.^18^

### Participant recruitment, consent and randomisation

Following ethical approval from Outer North London Research Ethics Committee (REC: 10/H0724/7), 20 participants, previously consented and block randomised in a clinical study investigating the effects of footwear on CLBP,^18^ were invited to take part by CSM. Inclusion criteria were: aged 18–65 years, with a 3-month or greater history of LBP. Exclusion criteria were as the main trial,^18^ excluding constant LBP, specific spinal diagnosis inappropriate for physiotherapy interventions (eg, spinal fracture of infection); any condition inappropriate for exercise physiotherapy (eg, severe cardiovascular or metabolic disease) or for wearing rocker-sole footwear (eg, Morton's neuroma, peripheral neuropathy); and participants who had previously used rocker-sole shoes.

### Interventions

**Figure 1:**
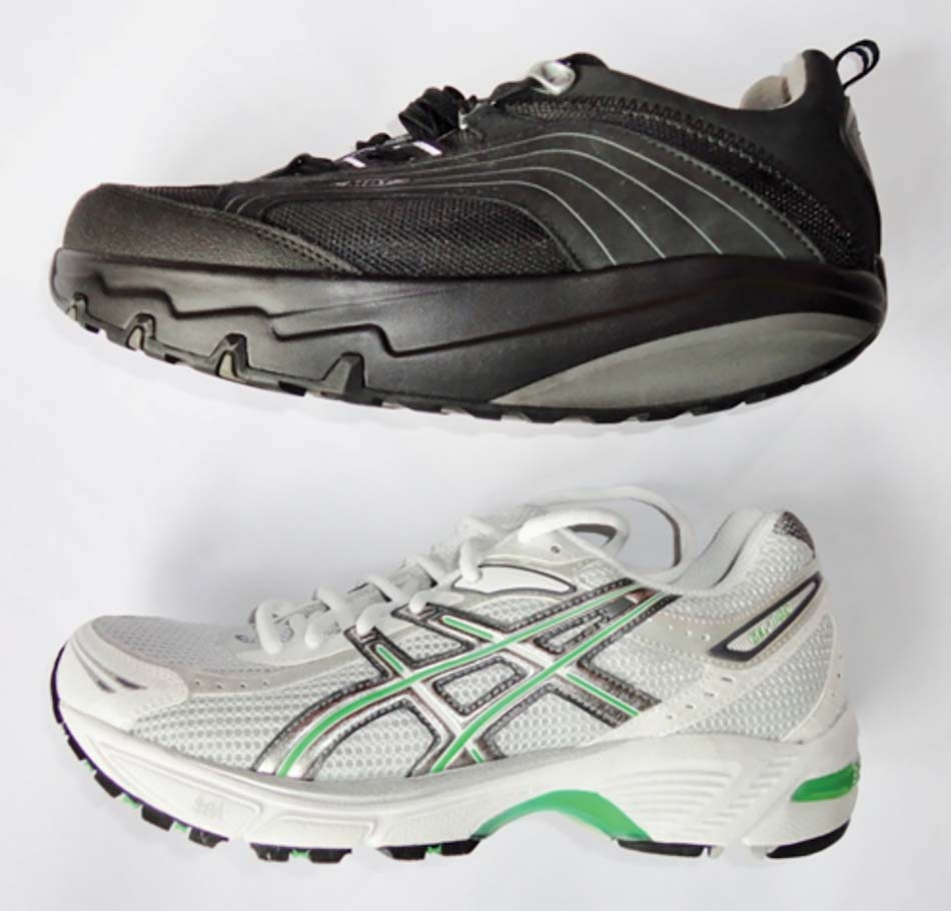
Study shoes: rocker-sole shoe (top); flat-sole shoe (bottom).

On consenting and entering the current study, participants were already allocated either the rocker-sole (Masai Barefoot Technology (MBT) Chapa Caviar, Masai GB Limited, London, UK) or the flat-sole shoe (Gel 1140, ASICS, Warrington, UK) (figure 1).^18^

Participants had been fitted with their allocated footwear type and taught how to walk in their shoes (see online supplementary appendix 1). They were instructed not to wear their allocated shoes prior to baseline biomechanical assessment, then wear them for a minimum of 2 hours/day while standing or walking for the study duration. Between baseline and 6-week assessment, participants attended a 4-week LBP exercise group (fulfilling methods of the main clinical study participants were recruited from^18^).

10.1136/bmjsem-2016-000170.supp1 supplementary appendix

### Data collection

Data collection occurred at the ‘One Small Step Gait Laboratory’, Guys' Hospital, London. Demographic, back-pain disability (Roland-Morris Questionnaire) and pain scores (numerical rating scale) were recorded at baseline.

#### Biomechanical assessment

Participants were assessed wearing short trousers and vest or no top. Participants' anthropometric measurements (pelvic width; leg length; knee width; ankle width; height; and weight) were recorded to inform the mechanical model formulated for each participant in Vicon's Nexus (1.8.1) motion capture software (Vicon Motions systems, Oxford, UK).

**Figure 2:**
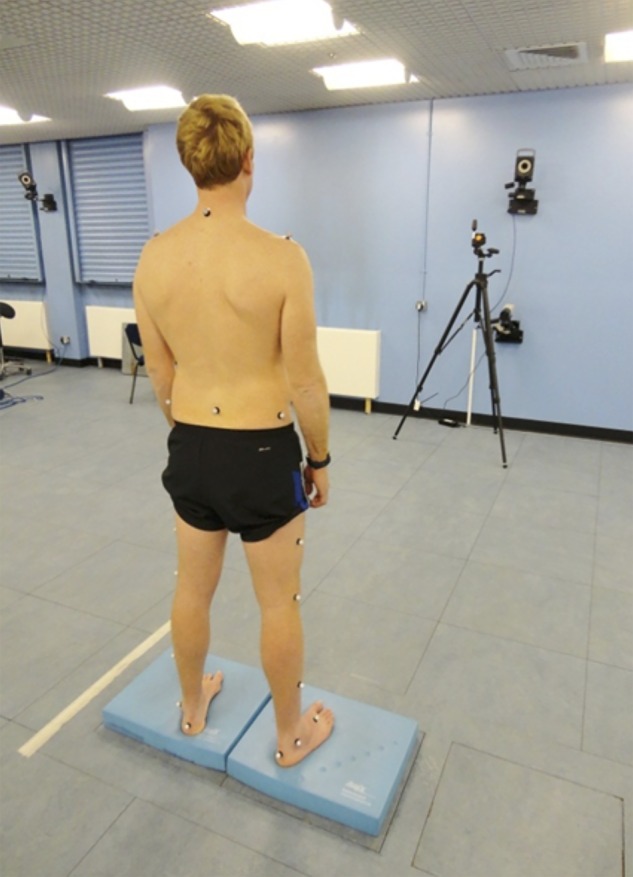
Participant standing on foam cushions overlying force plates.

Participants were assessed barefoot and shod, with their feet on adjacent force plates (FP5000, AMTI, Massachusetts, USA), during four posture-challenging standing conditions involving manipulation of visual input and support surface: (1) firm surface, eyes-open; (2) firm surface, eyes-closed; (3) compliant surface, eyes-open; (4) compliant surface, eyes-closed. Compliant surface was achieved by placing an Airex™ cushion (48.5×40.0×6.4 cm, 0.7 kg, high density (50 kg/m^3^), closed-cell foam) (l-group, St Louis, Missouri, USA) over each force plate (figure 2).

##### Barefoot assessment

Participants stood barefoot, feet approximately pelvis width apart and were instructed to keep their eyes focused on a red sticker at eye height on a tripod 3 m in front of them.^19^ Participants were assessed for three 40 s trials (shown to produce acceptable reliability^20^) for each standing condition. The middle 30 s of each trial was analysed to avoid possible initial sway errors and effects of participant fatigue or anticipation of a trial ending.

Each participant received the same instructions at the start of each trial:

When I say ‘Go’ I want you to stand and maintain your balance until you hear the instruction to rest. Each trial will last for 40 seconds. Focus on the red sticker on the tripod ahead of you. Keep your arms relaxed by your sides.

A rest period of 20 s occurred between each 40 s trial. Sufficient trials were performed to enable three valid sets of data to be recorded. A test was invalidated if the participant: (1) moved their foot position during the test; (2) changed their arm starting position or (3) opened their eyes during an eyes-closed task.

##### Shod assessment

Study shoes were then put on. The shod assessment protocol was conducted as described in the *Barefoot assessment* section. Shod assessment protocol was conducted by AS; shoes were concealed from CSM to maintain assessor blinding in the main trial.^18^

### Outcome measures

The following postural stability primary outcomes were assessed at baseline, 6 weeks and 6 months: (1) root mean squared error and (2) velocity of the CoP in the anteroposterior direction (CoP_RMSE AP_ and CoP_VEL AP_, respectively). Equations, demonstrating how CoP data were calculated, are presented in online supplementary appendix 2.

10.1136/bmjsem-2016-000170.supp2 supplementary appendix

### Sample size

A sample size calculation was not conducted due to the lack of reported data of minimal clinically important difference for the primary outcome measures (CoP parameters).

### Data extraction

Industry-standard motion capture files (.c3d) containing force data were extracted. Force plate data were filtered with a low-pass (10 Hz) Butterworth filter. CoP parameters (CoP_RMSE_ _AP_ and COP_VEL AP_) were calculated using a proprietary program writer Visual Basic for Application (Microsoft Excel, Reading, UK).

### Data analysis

The primary analysis was by intention-to-treat, including all eligible randomised participants who provided follow-up data. Two-way mixed model (between–within) analysis of variances were conducted with one within-subject (assessment time points) and one between-group factor (footwear type) to compare the influence of footwear type over time and one within-subject (standing condition) and one between-group factor (footwear type) to compare baseline data between groups. Analysis of variance used data from participants with full data sets (rocker-sole group n=13, flat-sole group n=7 for baseline comparisons and immediate effect of footwear; rocker-sole group n=11, flat-sole group n=5 for long-term follow-up). Macuhly test of sphericity assumption and Levene's test of equality of variances assumption were considered for within-subject and between-subject effects, respectively. The α level for determining statistical significance was set at 0.05. Data were analysed using IBM SPSS V.20.0.0 (IBM, Armonk, New York, USA). Results are presented as means (SDs) unless otherwise stated.

## Results

Twenty participants (from 38 who showed interest in the study) were recruited into the study from June 2010 to November 2010 (the final 6 months of main study recruitment^18^). Seven participants had been prerandomised to receive the flat-sole and 13 to receive the rocker-sole shoe.^18^ There were no differences between the groups in demographic or outcome measures (table 1) at baseline.

**Table 1:** Baseline characteristics of the study participants

	Flat-sole group (n=7)	Rocker-sole group (n=13)	p Value
Gender			
Male	3 (42.9%)*	6 (46.2%)*	0.89†
Female	4 (57.1%)*	7 (53.8%)*	
Age (years)	37.9 (13.0)	42.6 (12.5)	0.43
Weight (kg)	82.4 (22.0)	70.3 (11.3)	0.12
Height (cm)	173.8 (7.3)	173.5 (9.5)	0.95
Roland Morris Disability Questionnaire (0–24; 0=best)	7.9 (1.8)	5.7 (3.3)	0.13
Numerical rating score for pain (0–10; 0=best)	6.3 (1.5)	5.7 (1.7)	0.48

Summary measures represent means (SD).

*Summary measures represent numbers (percentages).

†Data analysed with independent t-test or χ^2^ test.

Baseline barefoot CoP parameters are presented in table 2. There were no differences between the groups in CoP_RMSE AP_, CoP_VEL AP_ for any of the four standing conditions (F(3,51)=0.31, p=0.82, η^2^=0.02; F(1.76,29.94)=0.15, p=0.83, η^2^=0.01, respectively).

**Table 2:** Barefoot anteroposterior centre of pressure and postural strategy parameters at baseline

Standing condition	Group	CoP_RMSE AP_ (mm)	CoP_VEL AP_ (mm/s)
Eyes open firm surface	Flat-sole shoe	4.80 (2.47)	7.33 (2.01)
	Rocker-sole shoe	4.39 (1.84)	7.19 (1.13)
Eyes closed firm surface	Flat-sole shoe	4.98 (1.87)	7.54 (1.44)
	Rocker-sole shoe	4.05 (1.26)	7.50 (1.12)
Eyes open compliant surface	Flat-sole shoe	10.06 (2.87)	11.89 (1.18)
	Rocker-sole shoe	8.63 (2.61)	12.67 (4.38)
Eyes closed Compliant surface	Flat-sole shoe	11.06 (2.86)	17.94 (4.32)
	Rocker-sole shoe	10.62 (2.66)	17.75 (4.12)

Summary measures represent means (SD).

AP, anteroposterior; RMSE, root mean squared error; VEL, velocity.

**Figure 3:**
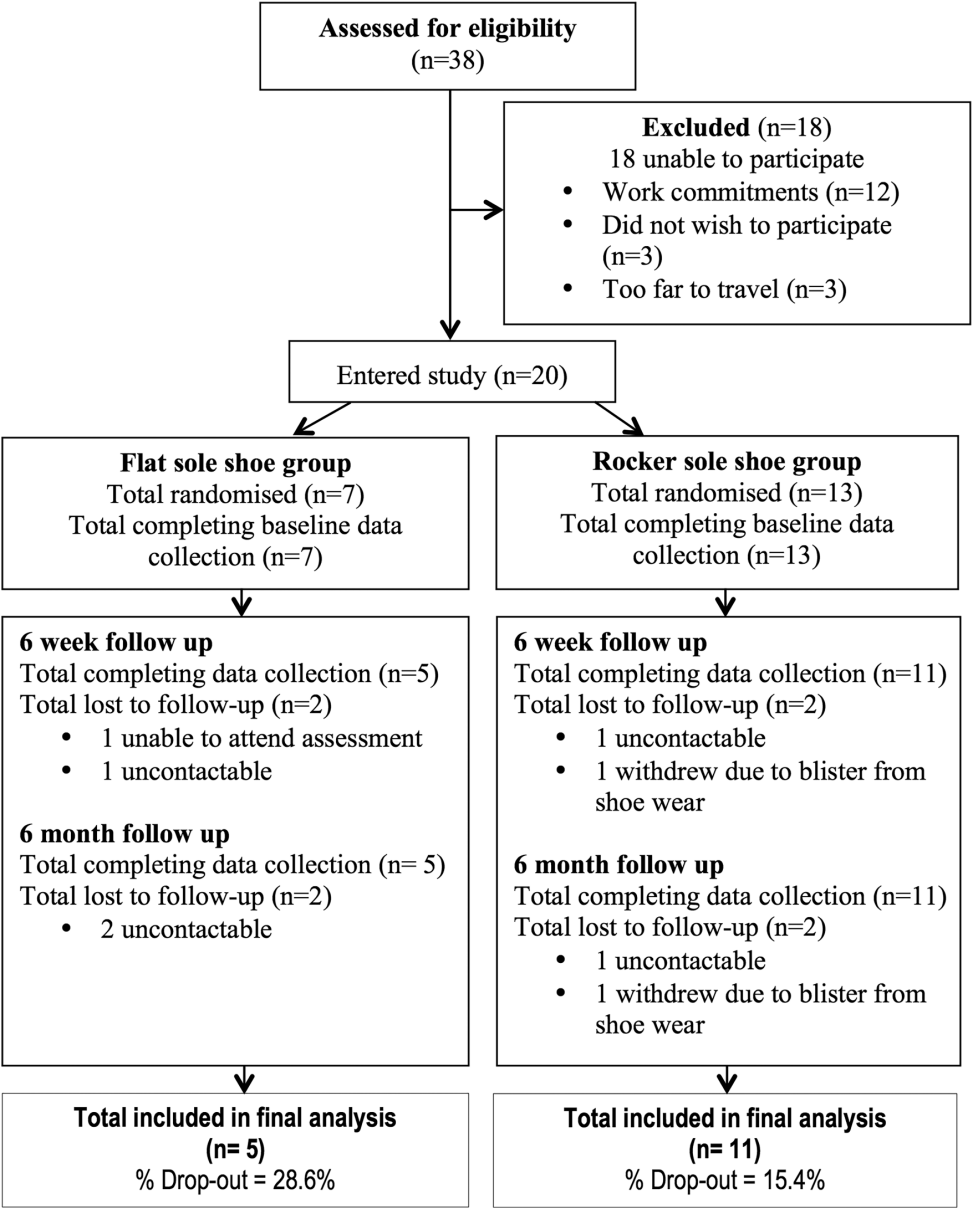
Flow of participants through trial.

Participant attrition and retention during the study are presented in figure 3. At 6 months, 16 (80%) participants were reassessed.

### Comparison of CoP parameters when standing barefoot and standing shod

Standing in rocker-sole shoes, with eyes-open on firm surface, resulted in a mean increase in CoP_RMSE AP_ of 6.41 mm (t(12)=7.77, p<0.01) and CoP_VEL AP_ of 4.10 mm/s (t(12)=7.14, p<0.01) when compared with standing barefoot (table 3). There was no difference in CoP_RMSE AP_ or CoP_VEL AP_ when standing in flat-sole shoes compared with barefoot (table 3).

**Table 3:** Sagittal plane centre of pressure parameters during barefoot and shod standing, with eyes open on firm surface

	Flat-sole shoe group (n=7)		Rocker-sole shoe group (n=13)	
	CoP_RMSE AP_ (mm)	CoP_VEL AP_ (mm/s)	CoP_RMSE AP_ (mm)	CoP_VEL AP_ (mm/s)
Barefoot	4.78 (2.26)	7.03 (2.00)	4.39 (1.84)	7.19 (1.13)
Shod	5.61 (2.33)	7.11 (1.27)	10.79 (3.01)	11.28 (1.93)
Difference between means	0.84 (2.03)	0.07 (1.20)	6.41 (2.97)*	4.10 (2.07)*

Summary measures represent means (SD) or percentages where indicated (%).

*Significant difference within groups between barefoot and shoe conditions (p<0.01).

AP, anteroposterior; RMSE, root mean squared error; VEL, velocity.

### Influence of long-term shoe wear on barefoot sagittal plane CoP parameters

Neither the rocker-sole nor the flat-sole group demonstrated change in CoP_RMSE AP_ or CoP_VEL AP_ when assessed barefoot during the most challenging standing condition (eyes-closed, compliant ground), at any follow-up point (rocker-sole group F(2,20)=2.28, p=0.13, η^2^=0.19 and F(2,20)=2.69, p=0.09, η^2^=0.21, respectively; flat-sole group F(2,8)=1.89, p=0.21, η^2^=0.32 and F(2,8)=0.27, p=0.70, η^2^=0.06, respectively) (table 4). Furthermore, there were no differences between-groups in CoP_RMSE AP_ or CoP_VEL AP_ at any follow-up point during the most challenging standing condition (F(2,28)=1.80, p=0.19, η^2^=0.11 and F(2,28)=0.28, p=0.76, η^2^=0.02).

**Table 4:** Change in barefoot centre of pressure parameters during standing, eyes closed on compliant surface at reassessment points

		Assessment			
	Centre of pressure parameter	Baseline	6 weeks	6 months	p Value
Flat-sole shoe group (n=5)	CoP_RMSE AP_ (mm)	10.80 (2.85)	10.70 (3.40)	9.29 (1.95)	0.21
	CoP_VEL AP_ (mm/s)	21.61 (3.48)	20.66 (4.82)	20.19 (5.85)	0.70
Rocker-sole shoe group (n=11)	CoP_RMSE AP_ (mm)	10.43 (2.85)	9.35 (2.62)	9.75 (3.08)	0.13
	CoP_VEL AP_ (mm/s)	17.85 (4.59)	15.28 (3.64)	15.77 (4.26)	0.09

Summary measures represent means (SD).

No difference in COP_RMSE AP_ or CoP_VEL AP_ was found for the three less challenging standing conditions assessed within-shoe or between-shoe groups at any follow-up point.

### Influence of long-term shoe wear on postural control assessed when shod

When standing in study shoes, with eyes-open on firm surface, no significant differences were observed in CoP_RMSE AP_ or CoP_VEL AP_ for either shoe group at any reassessment point (rocker-sole group: F(2,20)=1.35, p=0.28, η^2^=0.12, and F(2,20)=1.84, p=0.19, η^2^=0.15, respectively; flat-sole group: F(2,8)=0.74, p=0.51, η^2^=0.16, F(2,8)=0.63, p=0.56, η^2^=0.14). Furthermore, while wearing study shoes, there were no differences between-groups in change in CoP_RMSE AP_ or CoP_VEL AP_ at any reassessment point (F(2,28)=1.18, p=0.32, η^2^=0.08, and F(2,28)=0.37, p=0.70, η^2^=0.03, respectively) (table 5).

**Table 5:** Change over time in anteroposterior centre of pressure parameters during shod standing, eyes open on firm surface

		Assessment			
	Centre of pressure parameter	Baseline	6 weeks	6 months	p Value
Flat-sole shoe group (n=5)	CoP_RMSE AP_ (mm)	5.20 (1.52)	6.03 (2.95)	5.29 (2.22)	0.51
	CoP_VEL AP_ (mm/s)	7.28 (2.04)	6.22 (1.16)	6.29 (1.97)	0.56
Rocker-sole shoe group (n=11)	CoP_RMSE AP_ (mm)	10.17 (2.84)	9.54 (2.79)	11.07 (3.89)	0.28
	CoP_VEL AP_ (mm/s)	9.39 (2.24)	9.10 (3.25)	8.24 (1.81)	0.19

Summary measures represent means (SD).

## Discussion

This study investigated the influence of rocker-sole shoes on postural stability in people with CLBP. The results were concordant with Hypothesis 1; that is, that the wearing of rocker-sole shoes provides a less stable surface to stand on than flat-sole shoes. However, the results do not support Hypothesis 2; there were no differences in barefoot CoP parameters within-groups or between-groups during barefoot trials at 6 weeks or 6 months, compared with baseline, for any standing condition. Furthermore, there were no changes from baseline in CoP parameters in the rocker-sole group when shod at 6 weeks and 6 months. These findings suggest that adaptation of the postural control system did not occur following long-term wear of rocker-sole shoes. Alternatively, the outcomes assessed were not appropriate to detect any potential training effect offered by the rocker-shoes.

### Anteroposterior CoP parameters

The current study demonstrated similar barefoot baseline CoP parameters between shoe groups. When compared with the findings of other studies investigating CLBP with the same outcome measures under similar protocols, this study demonstrated increased postural stability during less challenging standing conditions,^6^ ^11^ ^21^ and reduced postural stability during more challenging standing conditions.^11^ ^21^ ^22^ These differences may be due to a number of methodological and demographic differences reported to influence outcome, namely: number of trials;^10^ trial durations;^10^ participant age;^23–26^ body weight;^27^ ^28^ body height^27^ ^28^ and gender.^25^ However, the consistent increase in CoP parameters from stable to more challenging standing conditions in the current study concurs with other studies.^7^ ^11^

A reduction in a CoP parameter is interpreted as an improvement in postural stability.^10^ It was hypothesised that due to the increased proprioceptive input from wearing rocker shoes,^12^ a greater reduction in barefoot and shod postural excursion may occur at reassessment in the rocker-sole compared with the flat-sole group. However, neither group demonstrated a significant change in CoP parameters at any follow-up compared with baseline when barefoot or shod. This lack of change suggests that the rocker-sole footwear either (1) provided an additional postural challenge; however, the type of challenge did not result in long-term improvements in sensorimotor function, (2) provided an appropriate postural challenge but ‘dosage’ was insufficient for a training effect to occur or (3) influenced proprioceptive deficits; however, improvements were not detected.

The first explanation, suggesting that the increased postural challenge from rocker-sole shoes does not influence long-term improvements in sensorimotor function compared with wearing flat-sole shoes, concurs with the findings of other studies.^29^ ^30^ Nigg *et al*^29^ investigated the influence of rocker-sole footwear on balance in golfers with LBP and in people with knee osteoarthritis.^30^ In support of the current study findings, Nigg *et al*^29^ concluded that no differences in balance performance were detected between the intervention (rocker-sole group) or control group (normal shoes) at 6 and 12 weeks.^29^ The current study adds to Nigg *et al*'s conclusions by demonstrating that longer term use of rocker-sole shoes (6 months) has no further influence on postural stability.

The second explanation suggests a greater postural challenge may have resulted in a measured training effect. When compared with standing barefoot, the rocker shoes demonstrated a 57–146% increase in the CoP parameters assessed. Introducing additional postural challenge in an attempt to increase the CoP parameters further may not only be unsafe or impractical in a CLBP population, but may also, in the absence of evidence to support a relationship between increased postural challenge and change in CoP parameters or clinical change, be inappropriate.

The third explanation suggests that the null hypothesis was incorrectly accepted and study conclusions are incorrect. This may have been due to an underpowered sample, poor reliability of the outcome variables or an insensitivity to detect genuine changes in postural control. The reliability of the outcome variables may be improved by increasing the duration and number of trials. However, of the numerous CoP parameters regularly reported in research assessing postural stability, the two parameters chosen in the current study have been reported as highly reliable.^10^

Although changes in CoP parameters have been suggested as appropriate outcome measures to detect clinical change,^31^ to the authors knowledge, measurements of the SE of CoP parameters, during challenging standing conditions, have yet to be reported in the literature for people with CLBP. The differences in postural instability outcomes during challenging standing conditions for both shoes types in the current study are less than the reported SEs of the same CoP parameters assessed in reliability studies investigating elderly participants (who also demonstrate poor postural stability).^32^ Changes in CoP parameters following an intervention may be too small to reliably determine whether change in postural stability has occurred.

The clinical study investigating the effects of rocker-sole footwear on CLBP,^18^ from which the current participants were recruited, demonstrated clinically important statistically significant reductions in disability and pain (in rocker-sole and flat-sole shoe groups) at follow-up; however, the current study demonstrates no change in postural parameters. This study and the findings of Kuukkanen and Malkia^33^ (who in the presence of improvement in function in patients with LBP, found no improvement in postural stability at 6 months following an exercise intervention) suggest that CoP parameters may be insensitive to real changes in postural control or that there may be no significant changes in control. If the latter, the use of any mechanical indices as outcome measures would be inappropriate; if the former, alternative mechanical outcome measures need to be developed and tested.

### Limitations

A systematic review investigating acceptable reliability for CoP parameters in asymptomatic individuals, published subsequently to the start of the current study, recommended a minimum trial duration of 90 s—a greater duration than that applied in this clinical trial.^34^ However, in the current study, prolonged standing may have aggravated symptoms, and negatively influenced attrition rates.

The authors recognise the small sample size of this study may have resulted in a type II error. Although the study sample is small (n=20), when compared with participants in the clinical study^18^ from which study participants were recruited (n=115), there were similar reductions in pain and disability at 6-week and 6-month follow-up (disability: rocker-sole group F(2,106)=0.20, p=0.82, η^2^=0.001; flat-sole group, F(1.53,73.4)=0.24, p=0.73, η^2^=0.01; pain: rocker-sole group, F(1.70,90.10)=0.01, p=0.99, η^2^ <0.01; flat-sole group, F(2,96)=1.04, p=0.36, η^2^=0.02), suggesting that this subgroup was a representative sample of a larger CLBP population, hence reducing the likelihood of a type II error.

It is unclear what effect either shoe type may have on CoP parameters in people with more severe CLBP, greater postural instability at baseline or if worn for >6 months.

### Conclusions

This is the first randomised trial with long-term follow-up comparing the influence of rocker-sole and flat-sole shoes on standing CoP parameters in a CLBP population. Long-term use of rocker-sole or flat-sole shoes in addition to attendance to a 4-week exercise group does not appear to influence barefoot postural control, as determined by CoP parameters, during standing in people with CLBP.
