# Supplementary material for: Do rocker-sole shoes influence postural stability in chronic low back pain? A randomised trial
Source: BMJ Open Sport Exerc Med. 2016 Oct 19;2(1):e000170. doi: 10.1136/bmjsem-2016-000170 (PMC5125425; doi:10.1136/bmjsem-2016-000170)
Supplement: supplementary appendix [file bmjsem-2016-000170supp_appendix1.pdf]

Standing and walking instructions for study shoes

ROCKER SOLE SHOE (wording as recommended by shoe manufacturer)

Walking Technique:

"Begin by walking naturally on a flat surface."

Cues:

- (Good speed): "Walk at a brisk pace."

- (Short steps): "Shorten the stride length."

- (Good posture) "Walk with good posture…"

  - (Draw up crown of head): "Lengthen your spine by drawing the crown of your head up.)
  - (Proper gaze): "Look straight ahead."
  - (Tummy in): "Pull your lower abdominal muscles in to help activate your core stability."
  - (Shoulders back): "Bring your shoulders back and in line with your ears and hips."

- (Swing arms with proper trunk movement): "Gently swing your arms and move your torso as you walk."

- (Roll through): "Make sure you roll through the feet with every step."

  - (Demonstrate proper roll through)
  - (Demonstrate where the heel sensor should hit): "Your foot should contact at the heel. As your foot rolls through from heels to toes, roll through the centre of the foot. When your foot pushes off, the weight in your toes should be evenly distributed so that the pressure is from the middle rather than to one side."

Standing Exercise:

- (Foot position): "Stand with your feet parallel and shoulder width apart."

- (Rock through the feet): "Roll forwards and backwards through the feet and ankles…

- (Find the Pivot Area): "…feeling the pivot area just in front of the heels."

- (Heels and toes off floor): "Come to rest on the Pivot area. You should feel that your heels and toes are slightly off the floor."

- (Posture): "Draw up your posture, lengthening your spine, gently drawing your shoulders back and softening your knees."

Correction of Wrong Technique: "When wearing your shoes, certain mistakes can occur. Be aware that you are walking properly in your shoes to gain the maximum benefit."

- (Watching feet): "Do not watch your feet or the floor – look straight ahead when wearing your shoes."

- (Slouched posture): "Walk and stand with good posture at all times."

- (Poor core control): “Remember to engage your core muscles.”

- (Stiff arms): “Walk with a relaxed, natural gait letting your arms swing gently and your torso move.”

- (Over-pronation): “Do not let your feet roll in when you stand or walk.”

- (Flat foot-strike): “Roll through the foot to get the full benefit of the shoes.”

- (Pain): “Walking should feel comfortable and natural. If you have pain, stop and check your technique or consult with your physiotherapist.”

Fitting: “Always make sure your shoes are snug on the instep, tight on the heel and comfortable on the toes.”

- **Should not**: “Your shoes should not slip at all, squeeze or rub the toes, press on the tips of your toes, rub against the ankle bones or cause you any pain.

- **General medical precautions**: “When you first start wearing your shoes you may experience certain short-term effects. These include tingling in the feet and toes and general aches in the muscles. These effects normally stop within one to two weeks of wearing them.”

- **Build up the amount of time you wear your shoes**: Start wearing the shoes for 15 to 30 minutes per day building up daily over the first week to two hours**.** Progress only as your comfort allows, to wearing a minimum of two hours per day.

- **Comfort:** If you encounter any problems with wearing your shoes, please contact your physiotherapist to discuss.

FLAT SOLE SHOE (wording altered from rocker sole shoe instructions above to apply to the flat sole shoe)

Walking Technique:

"Begin by walking naturally on a flat surface."

Cues:

- (Good speed): "Walk at a normal pace."

- (Normal stride): "Walk with your normal stride length."

- (Good posture) "Walk with good posture…"

  - (Draw up crown of head): "Lengthen your spine by drawing the crown of your head up.)
  - (Proper gaze): "Look straight ahead."
  - (Tummy in): "Pull your lower abdominal muscles in to help activate your core stability."
  - (Shoulders back): "Bring your shoulders back and in line with your ears and hips."

- (Swing arms with proper trunk movement): "Gently swing your arms and move your torso as you walk."

- (Roll through): "Make sure you roll through the feet with every step."

  - (Demonstrate proper roll through)
  - (Demonstrate where the heel should hit): "Your foot should contact at the heel. As your foot rolls through from heels to toes, roll through the centre of the foot. When your foot leaves the floor, push off through your big toe"

Standing Exercise:

- (Foot position): "Stand with your feet parallel and shoulder width apart."

- (Heels and toes on floor): "You should feel that your weight is evenly distributed over both feet."

- (Posture): "Draw up your posture, lengthening your spine, gently drawing your shoulders back and softening your knees."

Correction of Wrong Technique: "When wearing your shoes, certain mistakes can occur. Be aware that you are walking properly in your shoes to gain the maximum benefit."

- (Watching feet): "Do not watch your feet or the floor – look straight ahead when wearing your shoes."

- (Slouched posture): "Walk and stand with good posture at all times."

- (Poor core control): "Remember to engage your core muscles."

- (Stiff arms): "Walk with a relaxed, natural gait letting your arms swing gently and your torso move."

- (Over pronation): "Do not let your feet roll in when you stand or walk."

- (Flat foot-strike): “Roll through the foot to get the full benefit of the shoes.”

- (Pain): “Walking should feel comfortable and natural. If you have pain, stop and check your technique or consult with your physiotherapist.”

Fitting: “Always make sure your shoes are snug on the instep, tight on the heel and comfortable on the toes.”

- **Should not**: “Your shoes should not slip at all, squeeze or rub the toes, press on the tips of your toes, rub against the ankle bones or cause you any pain.

- **General medical precautions**: “When you first start wearing your shoes you may experience certain short-term effects. These include tingling in the feet and toes and general aches in the muscles. These effects normally stop within one to two weeks of wearing them.”

- **Build up the amount of time you wear your shoes**: Start wearing the shoes for 15 to 30 minutes per day building up daily over the first week to two hours**.** Progress only as your comfort allows, to wearing a minimum of two hours per day.

- **Comfort:** If you encounter any problems with wearing your shoes, please contact your physiotherapist to discuss.
